# Pharmacokinetic simulations for remdesivir and its metabolites in healthy subjects and patients with renal impairment

**DOI:** 10.3389/fphar.2025.1488961

**Published:** 2025-03-27

**Authors:** Shengjie Zhang, Sunggyeol Jeong, Botao Jiang, Harvey Ho

**Affiliations:** ^1^ Auckland Bioengineering Institute, The University of Auckland, Auckland, New Zealand; ^2^ Xianning Central Hospital, The First Affiliated Hospital Of Hubei University Of Science And Technology, Xianning, Hubei, China

**Keywords:** remdesivir, pharmacokinetics, model, renal-impaired, mixed effects, COVID-19

## Abstract

**Introduction:**

Remdesivir (RDV) is used for treating COVID-19 patients. This study aims to utilize an *in silico* pharmacokinetics model to simulate the pharmacokinetics of RDV, its intermediate metabolites (IM), and nucleoside monophosphate (NUC) in both healthy individuals and patients with renal impairment.

**Methods:**

A system of six ordinary differential equations (ODEs) was developed to describe the concentration profiles of RDV, IM and NUC in both central and peripheral compartments, with metabolism assumed to occur in both. Parameter fitting was conducted using the Monolix software, incorporating renal impairment as a covariant in the mixed-effects model. The pharmacokinetic data was sourced from a recently published clinical trial involving healthy controls and patients with varying degrees of renal impairment, as well as a prior clinical report on a kidney transplant patient. Goodness-of-fit was assessed by comparing the observed data with the prediction results.

**Results:**

The simulations captured the key pharmacokinetic characteristics of RDV and its metabolites, including the rapid decline of RDV and IM during the first hour. The simulation results were in good agreement with the observed data, with most observations falling within the 90% confidence intervals.

**Conclusion:**

A mathematical model has been developed that effectively captures the main pharmacokinetic features of RDV and its primary metabolites in both healthy subjects and patients with varying degrees of renal impairment.

## 1 Introduction

Remdesivir (RDV), also known as GS-5743, is an antiviral medication initially developed for treating Ebola virus disease (Warren et al., 2016). However, it has demonstrated broad-spectrum antiviral activity, including effectiveness against coronaviruses such as the Middle East respiratory syndrome coronavirus (MERS) (Sheahan et al., 2020) and severe acute respiratory syndrome coronavirus (SARS) (Sheahan et al., 2017). Due to these properties, RDV became a key therapeutic option during the COVID-19 pandemic. In October 2020, the U.S. Food and Drug Administration (FDA) granted emergency use authorization for RDV as a compassionate drug for patients experiencing severe symptoms of COVID-19 (Grein et al., 2020). Despite its potential benefits, Remdesivir is contraindicated in patients with an estimated glomerular filtration rate (eGFR) of less than 30 mL/min, including those with end-stage renal disease (ESRD), due to concerns about renal toxicity (Sörgel et al., 2021).

**FIGURE 1:**
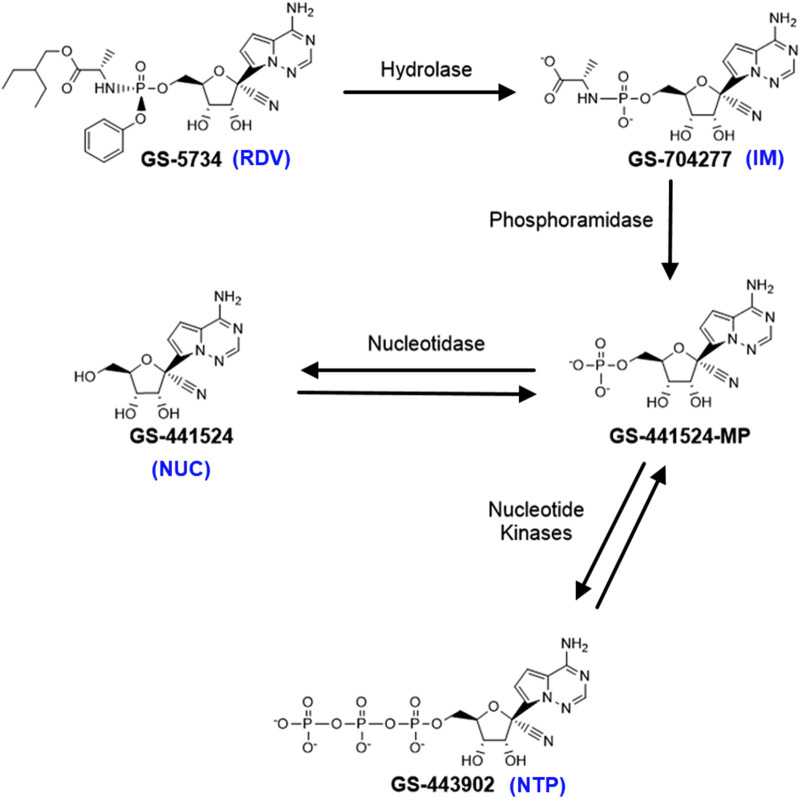
A brief diagram of the metabolic pathway of Remdesivir (GS-5734). Adopted from [6].

As a prodrug, RDV is metabolized into its active form, nucleoside triphosphate (NTP) GS-443902, which mimics adenosine. This active metabolite inhibits the replication of SARS-CoV-2 by binding to viral RNA-dependent RNA polymerases (RdRp), leading to premature termination of viral RNA synthesis (Warren et al., 2016). The metabolic pathway of RDV has been extensively studied and documented in the literature, e.g., in (Warren et al., 2016), (Humeniuk et al., 2020) (Yang, 2020). Briefly, RDV enters peripheral blood mononuclear cells (PBMCs) and undergoes hydrolysis by esterase to form a transient intermediate metabolite (GS-704277). This intermediate is further converted into a nucleoside monophosphate, which can either be transformed into the nucleoside metabolite GS-441524 or metabolized into the active antiviral nucleoside triphosphate by intracellular enzymes (Figure 1).

Previous *in vitro* and *in vivo* studies have provided valuable pharmacokinetic (PK) data for RDV. For instance, Warren et al. investigated the time-concentration profiles of RDV and its metabolites in male adult rhesus monkeys (Warren et al., 2016). They observed that after intravenous administration of a 10 mg/kg dose of RDV, GS-5734 exhibited a rapid plasma half-life with fast systemic elimination. *In vitro* data have also demonstrated the antiviral activity of RDV against SARS-CoV-2 (Wang M. et al., 2020). Furthermore, clinical trials reported that COVID-19 patients treated with RDV experienced a shorter median time to recovery (11 days) compared to those in the placebo group (15 days) (Grein et al., 2020). Brooks et al. (2024) compared the pharmacokinetics of RDV in pregnant and non-pregnant women with COVID-19. The phase I study of Zhang et al. (2020) found that remdesivir and its metabolites (GS-704277 and GS-441524) were generally safe and well-tolerated in participants with varying degrees of renal impairment, including those on dialysis, without requiring dose adjustments. Determining an optimal dosing regimen for patients across different ages, genders, and underlying conditions requires extensive PK data. However, collecting such data from patients is often limited by ethical considerations and high operational costs. In this context, *in silico* modeling, which predicts the time courses of RDV and its metabolites using established PK principles, emerges as a valuable tool.

Several *in silico* models for the PK of RDV have been developed. For example, Goyal et al. created a two-compartment model to simulate the temporal profiles of RDV and its active metabolite, nucleoside triphosphate, in rhesus monkeys (Goyal A. et al., 2020). However, this model did not include intermediate metabolites such as GS-704277 and GS-441524. Hanafin et al. extrapolated a mice model to humans using allometric scaling to investigate the lung tissue distribution of RDV (Hanafin et al., 2021), while Maharaj et al. estimated age-specific RDV clearance in a pediatric population (6,000 simulated children from birth to 18 years old) using allometric scaling, though they also did not address RDV metabolites (Maharaj et al., 2020). Fan et al. employed a physiologically based pharmacokinetic (PBPK) model to estimate the exposure of the active metabolite in the lungs and liver of patients with organ dysfunction (Fan et al., 2022). Their simulations suggested a slight increase in GS-443902 exposure in the liver of renal-impaired subjects, with no impact in the lungs. However, this study did not incorporate clinical data from renal-impaired patients. Similarly, Abouellil et al. used a compartmental model to perform population PK analysis for RDV and its metabolites in healthy subjects, without accounting for those patients with impaired renal functions (Abouellil et al., 2023).

The aim of our work is to construct a mathematical model that includes both RDV and its major metabolites, such as GS-704277 and GS-441524, and to incorporate pharmacokinetic data from the recent Phase I clinical trial by Zhang et al. (2020), which studied patients with varying degrees of renal impairment. By comparing key parameters of the model in patients with and without renal impairment, we aim to gain insights into the PK behavior not only in the general population, but also in these vulnerable populations such as those with ESRD. The following sections provide a brief description of our methodology and a presentation of the simulation results.

## 2 Methods

### 2.1 Published *in vivo* and clinical data for model construction

Published studies including PK data for RDV and its metabolites are limited. In this study, we primarily utilized data from two key sources. The first source, i.e., of Zhang et al. (2020) is a phase I, open-label, parallel-group study included participants with mild (n = 12), moderate (n = 11), or severe (n = 10) renal impairment, as well as those with kidney failure (n = 6 on dialysis, n = 4 without dialysis). The renal impairment stages were classified according to: mild (60–89 mL/min/1.73 m^2^), moderate (30–59 mL/min/1.73 m^2^), severe (15–29 mL/min/1.73 m^2^), and kidney failure (<15 mL/min/1.73 m^2^). Healthy matched controls served as the reference group. The second source is a case study detailing the PK of RDV and its metabolites in a male patient in his mid-seventies diagnosed with COVID-19 (Sörgel et al., 2021). This patient, who was receiving renal replacement therapy, had an eGFR of 0 mL/min, indicating the absence of residual renal function (Sörgel et al., 2021).

The pharmacokinetic profiles of RDV and its metabolites, as reported in the literature, were digitized using the open-source software Engauge Digitizer (Version 12.1). Due to the absence of individual patient-level data, only the mean drug concentration values from the profile curves were extracted.

### 2.2 Construction of an *in silico* PK model

**FIGURE 2:**
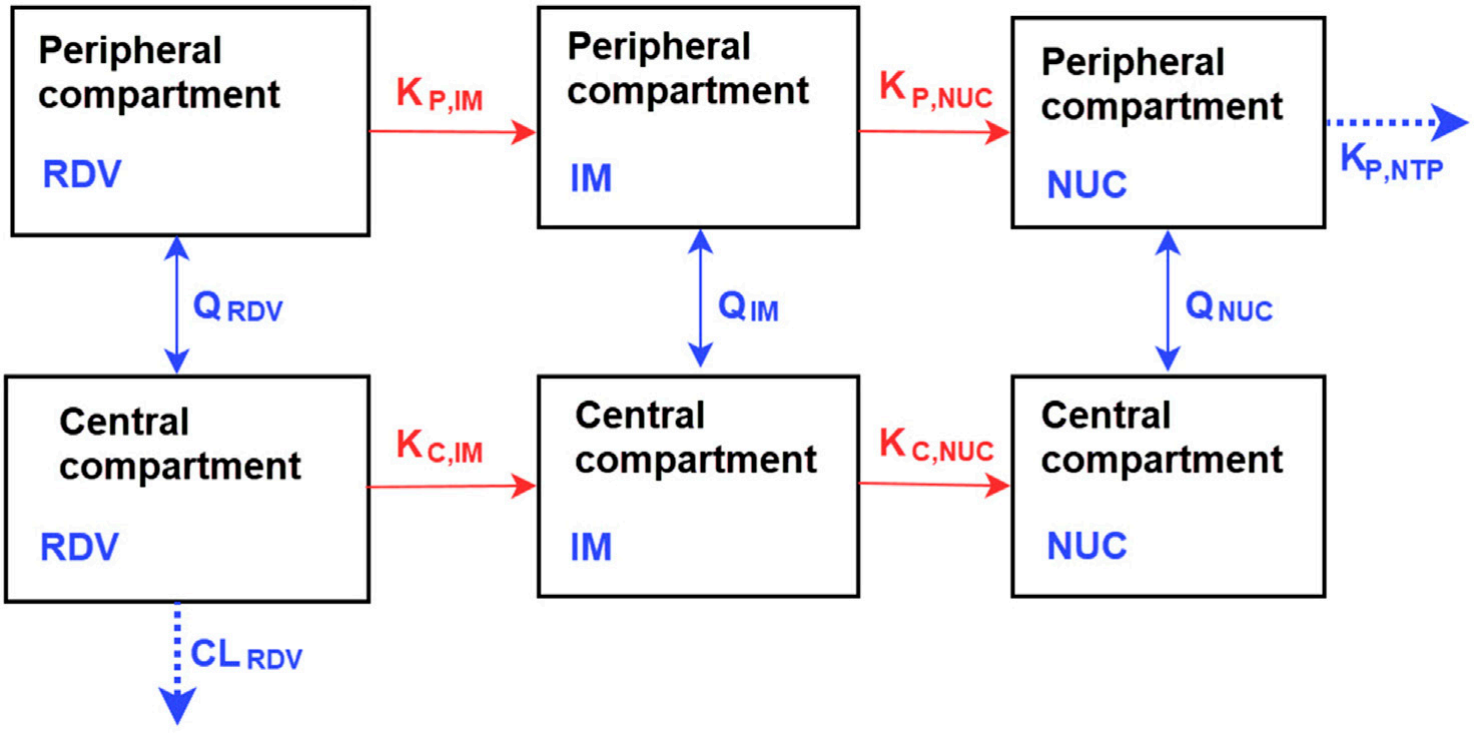
Representation of the compartmental model for the pharmacokinetics of RDV and its metabolites, the intermediate metabolites (IM), and nucleoside monophosphate (NUC). The model, based on the work of Abouellil et al. (2023) and with modifications, consists of central and peripheral compartments. Metabolism of RDV occurs in both compartments. More descriptions of the model are detailed in Methods.

To incorporate the above-stated data, we adopted a two-compartment mechanism-based model similar to that used in (Abouellil et al., 2023). As illustrated in Figure 2, the model tracks the concentrations of RDV, its intermediate metabolites (IM), and nucleoside monophosphate (NUC) in a series of central and peripheral compartments. The model assumes that metabolism primarily occurs in the central compartment, progressing sequentially from one compartment to the next. Additional metabolism was proposed to occur from the peripheral remdesivir compartment to its respective peripheral compartment, with elimination assumed to take place from the central compartment of RDV, and formation of the active GS-443902 in the peripheral compartment. The final model was selected based on goodness-of-fit plots, visual predictive checks, metabolic plausibility, parameter shrinkage by removing correlated parameters.

The model is governed by the following system of ordinary differential equations, representing metabolic rates and clearance variables for RDV, IM, and NUC:

$$\frac{dC_{C,RDV}}{dt}=Q_{RDV}\times \left(C_{P,RDV}-C_{C,RDV}\right)-K_{C,IM}\times C_{C,RDV}-{CL}_{C,RDV}\times C_{C,RDV}$$ (1)

$$\frac{dC_{P,RDV}}{dt}=Q_{RDV}\times \left(C_{C,RDV}-C_{P,RDV}\right)-K_{P,IM}\times C_{P,RDV}$$ (2)

$$\frac{dC_{C,IM}}{dt}=Q_{IM}\times \left(C_{P,IM}-C_{C,IM}\right)+K_{C,IM}\times C_{C,RDV}-K_{C,NUC}\times C_{C,IM}$$ (3)

$$\frac{dC_{P,IM}}{dt}=Q_{IM}\times \left(C_{C,IM}-C_{P,IM}\right)+K_{P,IM}\times C_{P,RDV}-K_{P,NUC}\times C_{P,IM}$$ (4)

$$\frac{dC_{C,NUC}}{dt}=Q_{NUC}\times \left(C_{P,NUC}-C_{C,NUC}\right)+K_{C,NUC}\times C_{C,IM}$$ (5)

$$\frac{dC_{P,NUC}}{dt}=Q_{NUC}\times \left(C_{C,NUC}-C_{P,NUC}\right)+K_{P,NUC}\times C_{P,IM}-K_{P,NTP}\times C_{P,NUC}$$ (6)

In above equations, the dependent variables represent the concentrations of RDV and its metabolites either in central ( $C_{C,i}$ ​) or in peripheral ( $C_{P,i}$ ​) compartments, where i represents RDV and its metabolites, the intermediate metabolites (IM) and nucleosides (NUC). $Q_{i}$ represents the intercompartmental clearance, and ${CL}_{i}$ represents the clearance of RDV in the central compartment. $K_{C,i}$ and $K_{P,i}$ denote the formation clearance of metabolite i in the central and peripheral compartment, respectively. The values of the parameters are detailed in Table 1.

**TABLE 1:** Parameters and their estimated values for Equations 1–6. The simulation results derived from these values are presented in the Results section.

Parameters	Description	Parameters for healthy control (Zhang et al., 2020)	Parameters for severe renal-impaired patients (Zhang et al., 2020)	Parameters for a renal-impaired patient with eGFR = 0 (Sörgel et al., 2021)	Units
$Q_{RDV}$	Intercompartment clearance of RDV	0.19	0.19	0.31	/h
$Q_{IM}$	Intercompartment clearance of IM	12.53	12.33	83.8	/h
$Q_{NUC}$	Intercompartment clearance of NUC	0.039	0.038	50.02	/h
$K_{P,IM}$	Conversion rate from RDV to IM in the peripheral compartment	0.31	0.19	0.22	/h
$K_{P,NUC}$	Conversion rate from IM to NUC in the peripheral compartment	2.44	0.5	0.15	/h
$K_{P,NTP}$	Conversion rate from NUC to NTP in the peripheral compartment	3.28E10	8.08E10	0.037	/h
$K_{C,NUC}$	Conversion rate from IM to NUC in the central compartment	0.38	0.85	6.27	/h
$K_{C,IM}$	Conversion rate from RDV to IM in the central compartment	0.22	0.19	0	/h
${CL}_{C,RDV}$	Clearance of RDV	2.99	2.99	2.3	/h

At this stage we assume the transfer and clearance kinetics for RDV and its methabolites are linear. While this assumption is not entirely accurate, it significantly simplifies the model, and hence is commonly used in most *in silico* models for RDV. It is also worth noting that in literatures (Warren et al., 2016), (Sörgel et al., 2021) and (Humeniuk et al., 2020), the unit for concentrations were different. While µM was used in (Warren et al., 2016), ng/mL was used for (Siegel et al., 2017), (Humeniuk et al., 2020), and (Zhang et al., 2020). Due to RDV’s low oral bioavailability, the drug is primarily administered intravenously (Humeniuk et al., 2020) (Abouellil et al., 2023) (Siegel et al., 2017).

### 2.3 Mixed effect model and covariant analysis

Equations 1–6 form the structural model for the pharmacokinetics of RDV and its metabolites. Random effects were assigned to all parameters with lognormal distributions. The combined error model was used to match observations (Equation 7):

$$C_{ij}=Y_{ij}+\sqrt{a^{2}+{\left(b\times Y_{ij}\right)}^{2}}$$ (7)

where $C_{ij}$ and $Y_{ij}$ are the observations and predictions for species i at time j . a and b are additive and proportional errors, respectively (Abouellil et al., 2023).

Renal impairment was included as a categorical covariate in the model, with 0 representing controls and 1 indicating renal impairment. To maintain simplicity, varying degrees of renal impairment were not further distinguished as separate covariates. The stochastic approximation expectation maximisation (SAEM) algorithm in the Monolix software (Lixoft, France) was used to perform parameter estimation.

## 3 Results

### 3.1 Clinical study based simulation 1

**FIGURE 3:**
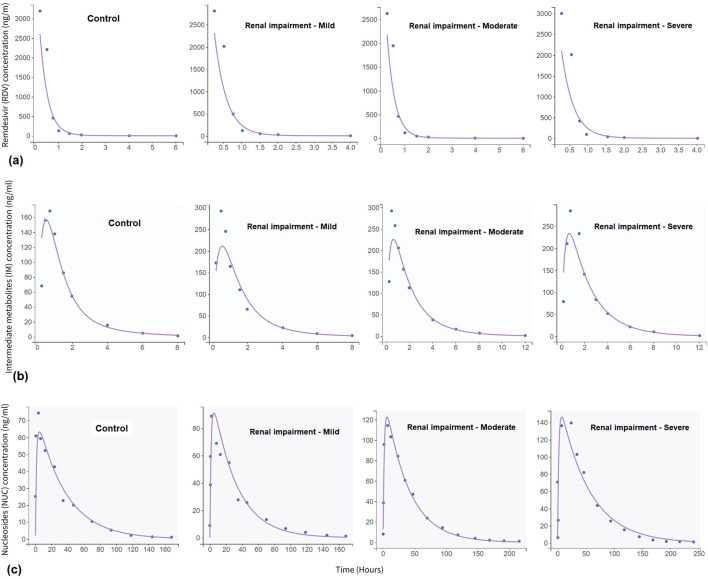
Simulated time-concentration profiles for: **(a)** remdesivir (RDV), **(b)** intermediate metabolites (IM), and **(c)** nucleoside monophosphate (NUC). The concentration of RDV drops sharply within the first 1 h, then forms a much more clearance profile. The data, represented by blue dots, were digitised from the clinical trial described in Zhang et al. (2020).

**FIGURE 4:**
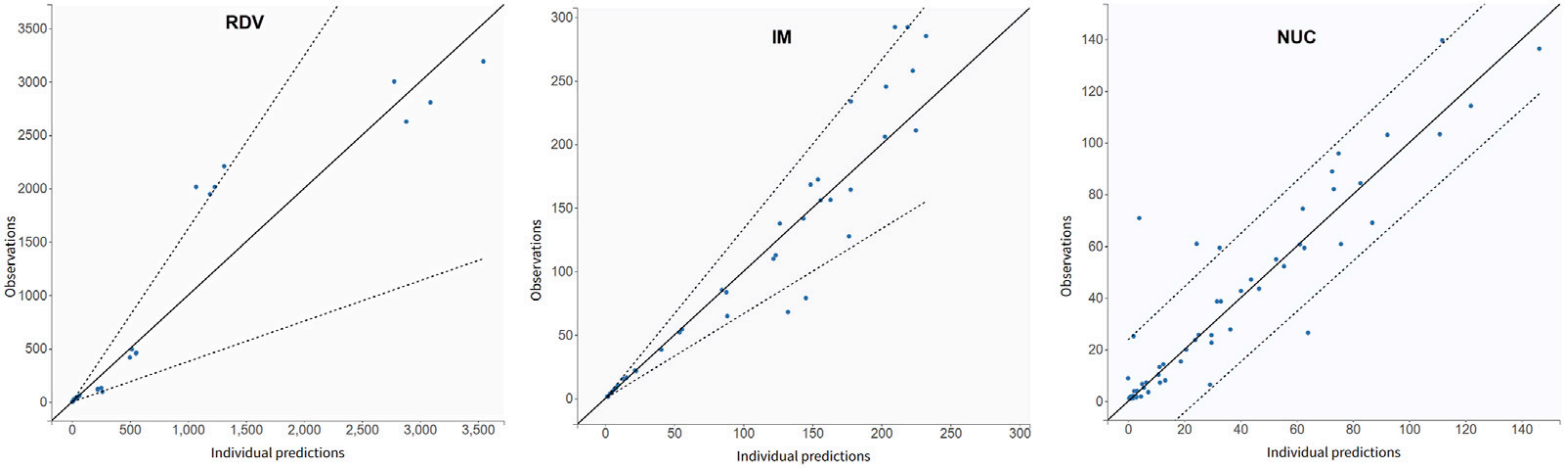
Observations (represented by blue dots) vs. simulation results as a metric for goodness-of-fit. The dotted lines represent 90% confidence intervals. The data were derived from the mean concentration values in Zhang et al. (2020) and do not differentiate between the control group and patients with renal impairment.

In the clinical trial conducted by Zhang et al. (2020), RDV was administered as single intravenous doses based on the degree of renal impairment: 100 mg for participants with mild or moderate impairment, 40 mg for those with severe impairment or predialysis kidney failure, and 20 mg for individuals with postdialysis or non-dialysis kidney failure. Figure 3 illustrates the simulated time-concentration profiles of RDV, IM, and NUC across the control group and various renal impairment cohorts, with data points representing mean concentrations rather than individual measurements. Figure 4 presents the observed versus predicted values from our model, with 90% prediction intervals (dotted lines) demonstrating the model’s goodness-of-fit.

The mixed-effects model indicates that the covariate “Renal Impairment” should be assigned to parameters $K_{C,IM}$ , $K_{P,IM}$ , and $K_{P,NUC}$ , highlighting their influence on the pharmacokinetics of RDV in healthy individuals and patients with renal impairment.

### 3.2 Clinical study based simulation 2

**FIGURE 5:**
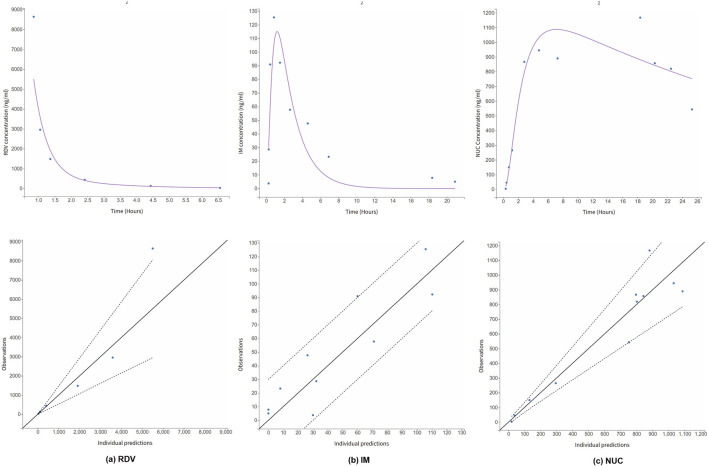
Simulated time-concentration profiles for **(a)** RDV, and its metabolites **(b)** IM and **(c)** NUC in an extreme case of renal impaired patient with eGFR = 0. The PK data was digitized from literature (Sörgel et al., 2021). Goodness-of-fit metric is also provided. The dotted lines are the 90% confidence intervals.

In the pharmacokinetic study of RDV in a kidney transplant recipient conducted by Sörgel et al. (2021), RDV was administered using a standard 5-day regimen. The treatment began with an initial 200 mg infusion on the first day, followed by daily 100 mg infusions over the next 4 days. For model validation, we specifically analyzed data from the first day of administration. Figure 5 presents the time-concentration profiles of RDV, IM and NUC, accompanied by the corresponding goodness-of-fit metrics. Notably, the model successfully captured the key pharmacokinetic characteristics of RDV and its metabolites, even in this extreme case of renal impairment with an eGFR of 0.

## 4 Discussion

During the COVID-19 pandemic, there was an urgent need for effective treatments for the rapidly increasing number of COVID-19 patients. Although RDV has been approved for compassionate use in treating COVID-19, its clinical efficacy remains controversial. For example, in a randomized, double-blind, placebo-controlled, multicenter trial conducted in China, RDV use did not correlate with the recovery time in 237 patients (Wang Y. et al., 2020). Yan and Muller have argued that GS-441524 may be a more suitable anti-COVID drug than Remdesivir due to its persistence in serum (Yan and Muller, 2020). Additionally, remdesivir can exhibit synergistic effects when co-administered with host-directed drugs such as dexamethasone (Yasuda et al., 2022), fluoxetine (Schloer et al., 2021), and baricitinib (Kalil et al., 2021). These drug-drug interactions (DDI) can influence its efficacy.

The primary novelty of this work lies in its simulations of the pharmacokinetics of RDV and its metabolites in healthy (control group) and patients of varying degrees of renal impairment, based on recently published Phase I clinical trial data (Zhang et al., 2020). It is worth mentioning that this PK study was performed by the company (Gilead Sciences Inc., USA) that developed RDV in the first place, and the PK data were collected from multiple medical centres in three countries. This study was also different from an earlier trial of the company that recorded the pharmacokinetic data of RDV and its metabolites in a cohort of healthy volunteers (Humeniuk et al., 2020).

Clinical data and our simulations reveal that the pharmacokinetics of RDV and its metabolites, IM and NUC, exhibit no significant differences across varying degrees of renal impairment, including in patients undergoing dialysis (Zhang et al., 2020). However, during parameter fitting, it became evident that renal impairment needs to be accounted for in the parameters $K_{C,IM}$ , $K_{P,IM}$ , $K_{P,NUC}$ . This suggests that the metabolism of RDV to IM in both central and peripheral compartments, as well as the conversion of IM to NUC in the peripheral compartment, is influenced by renal impairment. Clinically, this indicates that severe renal impairment, such as kidney failure, could adversely impact the metabolism of RDV to IM in plasma and the conversion of IM to NUC in peripheral blood mononuclear cells.

It is important to emphasize that various factors affect the PK profiles of RDV and its metabolites, and much data is still lacking. For instance, while RDV and NUC data were recorded in renal-impaired patients by Sörgel et al. (2021) and Zhang et al. (2020), pharmacokinetic data for NTP were not reported, highlighting the need for further validation of NTP concentration profiles. Additionally, metabolic differences among human subjects could influence the PK profiles of RDV. The results released by Gilead, the manufacturer of the drug, were based on Phase 3 trials of RDV in healthy subjects (Humeniuk et al., 2020). Patients with underlying conditions, such as liver disease, may have compromised metabolism and clearance functions. At the time of writing, there were still few reports on *in vivo* PK data for RDV in patients with impaired hepatic function. A recent study simulated the PK of RDV in hepatically impaired subjects (Fan et al., 2022); however, the data were derived from virtual patients.

**FIGURE 6:**
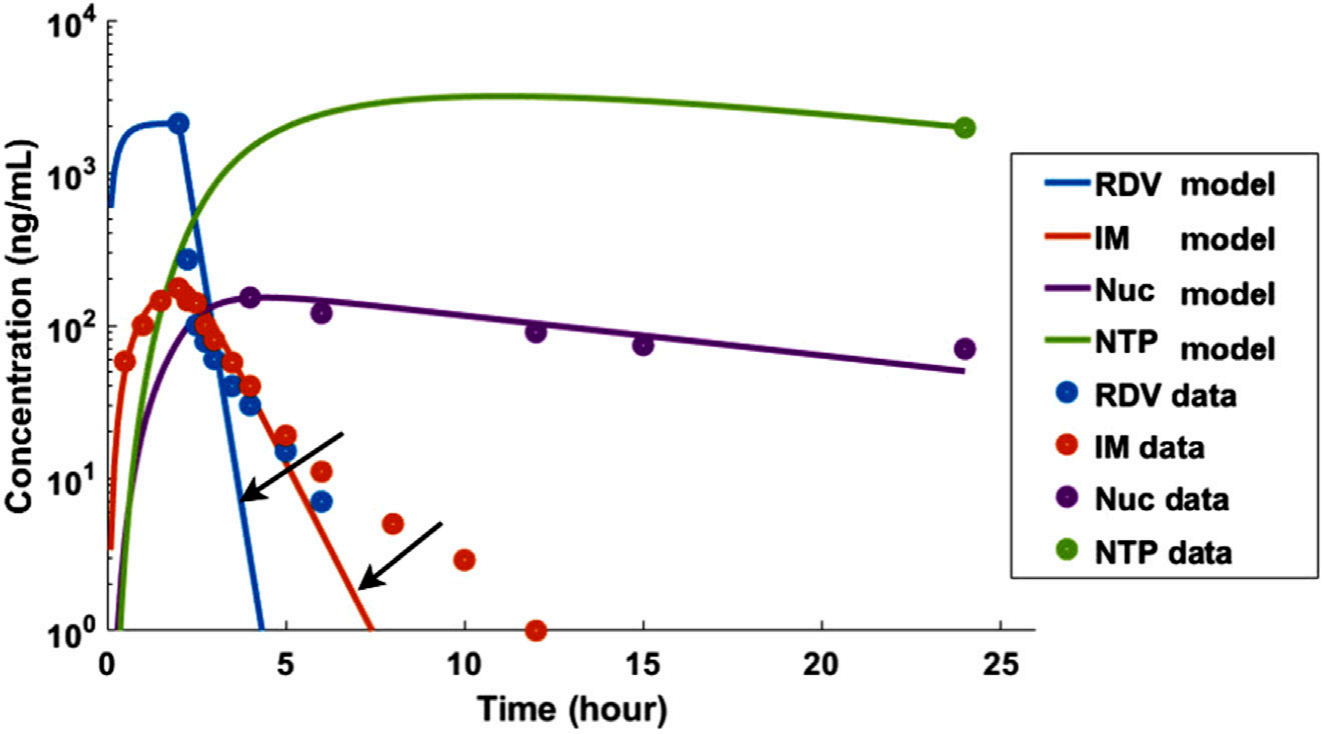
A simpler two-compartment model, similar to the one introduced by Goyal A. et al. (2020), was unable to adequately capture the two-phase decay of RDV and IM, as indicated by the two arrows. The pharmacokinetic data used in this analysis was based on Humeniuk et al. (2020).

Regarding the limitations of the model, firstly, while our simulations for RDV and its metabolites largely align with published data, our model is inherently more complex than the two-compartment model proposed by Goyal A. et al. (2020), requiring a greater number of parameters. The primary purpose of the more complex model was to address the issue of the two-phase slower decay observed after the initial rapid drop in concentrations for both RDV and IM, as illustrated in Figure 6. This was achieved by incorporating the assumption that metabolism of RDV and IM occurs in the peripheral compartment. Secondly, the clearance kinetics for RDV and its metabolites were assumed to be linear, yet a saturation mechanism could also occur, which would require Michaelis-Menten kinetics. However, this would again introduce more unknown parameters into the system, potentially rendering it over-parameterized.

It is important to recognize that different aspects of RDV pharmacokinetics may require distinct modeling approaches. For instance, while our model and the one presented by Deb and Reeves (2021)—a PBPK model implemented using GastroPlus—are both *in silico* models, they address separate aspects of RDV PK. Our model focuses on the pharmacokinetics of RDV and its metabolites (IM and NUC), whereas the work of Deb and Reeves (2021) emphasizes drug-drug interactions (DDI). A whole-body PBPK model incorporates numerous compartments for organs and tissues, necessitating a significantly larger number of physiological and drug-related parameters compared to our simpler model. Many of these parameters, such as the partition coefficients of RDV and its metabolites in various tissues, remain unknown and must be estimated or computationally fitted, along with uptake and clearance parameters. Commercial software like SimCyp and GastroPlus provides key advantages, including access to physiological parameters at the population level and chemical properties of compounds, which simpler models are unable to offer.

Future studies should aim to validate the pharmacokinetics of nucleotide triphosphate (NTP) in peripheral blood mononuclear cells under varying degrees of renal impairment and other pathological conditions, using a combination of clinical trials and *in silico* modeling. Additionally, the DDI features demonstrated in the work of Deb and Reeves (2021) provide valuable insights that could enhance the design of future clinical trials and modeling efforts.

## 5 Conclusion

In conclusion, this study introduces a mathematical model for the pharmacokinetics of remdesivir and its metabolites in both healthy individuals and patients with renal impairment. The simulated pharmacokinetic profiles demonstrate strong alignment with *in vivo* data from a Phase I clinical trial.

## Data Availability

The original contributions presented in the study are included in the article/Supplementary Material, further inquiries can be directed to the corresponding authors. The Matlab script for this model is available from the corresponding author HH upon request.
